# Exploring Attitudes, Subjective Norms and Perceived Behavioural Control in a Genetic-Based and a Population-Based Weight Management Intervention: A One-Year Randomized Controlled Trial

**DOI:** 10.3390/nu12123768

**Published:** 2020-12-08

**Authors:** Justine R. Horne, Jason A. Gilliland, Marie-Claude Vohl, Janet Madill

**Affiliations:** 1 Health and Rehabilitation Sciences, The University of Western Ontario, London, ON N6A 3K7, Canada; 2 The East Elgin Family Health Team, Aylmer, ON N5H 1K9, Canada; 3 Human Environments Analysis Laboratory, The University of Western Ontario, London, ON N6A 3K7, Canada; jgillila@uwo.ca; 4 Centre Nutrition, Santé et Société (NUTRISS) and Institute of Nutrition and Functional Foods (INAF), Laval University, Quebec, QC G1V 0A6, Canada; Marie-Claude.Vohl@fsaa.ulaval.ca; 5 Department of Geography, Western University, London, ON N6A 3K7, Canada; 6 School of Health Studies, Western University, London, ON N6A 3K7, Canada; 7 Department of Paediatrics, Western University, London, ON N6A 3K7, Canada; 8 Department of Epidemiology and Biostatistics, Western University, London, ON N6A 3K7, Canada; 9 Children’s Health Research Institute, London, ON N6A 3K7, Canada; 10 Lawson Health Research Institute, London, ON N6A 3K7, Canada; jmadill7@uwo.ca; 11 School of Food and Nutritional Sciences, Brescia University College, The University of Western Ontario, London, ON N6A 3K7, Canada

**Keywords:** theory of planned behaviour, theory of planned behavior, randomized controlled trial, behaviour change, behavioural determinants, nutrigenomics, nutrigenetics, lifestyle genomics, personalized nutrition

## Abstract

Background: Several studies demonstrate that the provision of personalized lifestyle advice, based on genetics, can help motivate individuals to engage in greater nutrition and physical activity changes compared to the provision of population-based advice. The theoretical mechanism behind this phenomenon is poorly understood. The objective of this study was to determine the impact of providing genetically tailored and population-based lifestyle advice on key constructs of the Theory of Planned Behaviour (TPB). Materials and Methods: A pragmatic, cluster randomized controlled trial (*n* = 140) took place at the East Elgin Family Health Team, in Aylmer, Ontario, Canada. Participants were primarily Caucasian females enrolled in a weight management program (BMI ≥ 25.0 kg/m^2^). Weight management program groups were randomized (1:1) to receive a population-based lifestyle intervention for weight management (Group Lifestyle Balance™ (GLB)) or a lifestyle genomics (LGx)-based lifestyle intervention for weight management (GLB+LGx). Attitudes, subjective norms and perceived behavioural control were measured at baseline, immediately after receiving a report of population-based or genetic-based recommendations and after 3-, 6- and 12-month follow-ups. Linear mixed models were conducted, controlling for measures of actual behavioural control. All analyses were intention-to-treat by originally assigned groups. Results: Significant changes (*p* < 0.05) in attitudes, subjective norms, and perceived behavioural control tended to be short-term in the GLB group and long-term for the GLB+LGx group. Short-term and long-term between-group differences in measures of subjective norms were discovered, favouring the GLB+LGx group. Conclusions: The TPB can help provide a theoretical explanation for studies demonstrating enhanced behaviour change with genetic-based lifestyle interventions. Clinical Trial Registration: NCT03015012.

## 1. Introduction

Behaviour change theories can help provide systematic explanations for why lifestyle-related behaviour change may or may not be observed. Established behavioural theory can and should be used to understand why certain interventions promote changes in lifestyle habits [1]. Several validated theories have been established to help predict human lifestyle-related behaviours [1]. The Theory of Planned Behaviour (TPB) is one of the most widely accepted behavioural theories, and suggests that attitudes, subjective norms and perceived behavioural control are significant predictors of one’s intention to engage in behaviours [2,3]. Attitudes are informed by behavioural beliefs or the beliefs about the likely consequences of a given behaviour. Subjective norms, or social pressures and behaviours, are informed by normative beliefs, which refer to beliefs about the typical habits and expectations of others. Control beliefs are considered to be perceptions about the presence or absence of factors that may facilitate or impede certain behaviours, and these control beliefs inform perceived behavioural control. Actual behavioural control is an important component of the TPB and refers to the skills, resources and other requirements that assist with or are needed to perform a behaviour. When a sufficient degree of actual behavioural control exists, intentions tend to be carried out [2,3]. Systematic reviews and meta-analyses have demonstrated that the TPB is a validated theory for predicting nutrition and physical activity (PA) intentions and resulting behaviours [1,4,5]. 

It is challenging to motivate lifestyle behaviour change in clinical practice [6,7], but several studies have demonstrated that providing genetic information and advice can help to motivate changes in nutrition and PA among patients [8,9,10]. This phenomenon has yet to be explained using the TPB [11], but researchers have called to action the incorporation of this theory into genetic testing behaviour change research [12]. Genetically tailored lifestyle advice can be referred to as lifestyle genomics (LGx), which is a science exploring interactions between genetics, lifestyle factors such as nutrition and physical activity (PA) and health outcomes [11]. For example, *FTO* genetic variation impacts weight loss responses to varying levels of PA [13]. Thus, genetic testing of *FTO* can provide guidance for genetically tailored PA targets. Similarly, different weight loss outcomes are observed in response to higher vs. lower protein dietary patterns, based on *FTO* genetic variation [14] and therefore genetic information can help to tailor nutrition plans. With this background in mind, the purpose of the present study was to determine the impact of providing genetically tailored and population-based lifestyle advice for weight management on key constructs of the TPB, while further comparing TPB changes between a group receiving population-based lifestyle advice and a group receiving genetic-based lifestyle advice. Overall, this study aimed to help elucidate a potential theoretical explanation for improvements in behaviour change resulting from genetic interventions.

## 2. Materials and Methods

Data Availability Statement: The dataset analysed is available from the corresponding author upon reasonable request.

**Figure 1:**
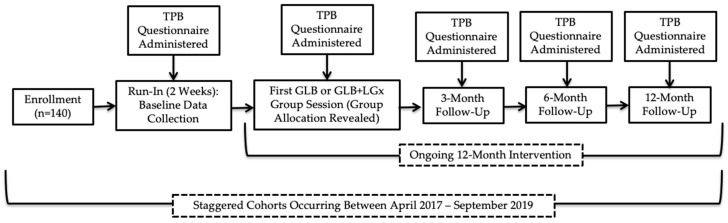
Flow of study design. TPB: Theory of Planned Behaviour; GLB: Group Lifestyle Balance™; LGx: lifestyle genomics.

This study was approved by the Western University Research Ethics Board (#108511). All participants provided written informed consent. Figure 1 provides a visual representation of the flow of the study (Clinical Trial Registration: NCT03015012), in which data collection occurred at baseline during a two-week run-in period, immediately after the first group session, and after 3, 6 and 12 months. Recruitment occurred between April 2017 and September 2018, with staggered intervention cohorts occurring from May 2017 to September 2019; the trial ended in September 2019 as the sample size was exceeded with 10 randomized groups. The CONSORT checklist was used to guide manuscript development; a CONSORT flow diagram has been previously published elsewhere [15]. The Nutrigenomics, Overweight/Obesity and Weight Management (NOW) Trial was a parallel-group, pragmatic, cluster randomized controlled trial (RCT), which was incorporated into the Group Lifestyle Balance™ (GLB) program. GLB groups were randomized 1:1 to receive either the standard population-based intervention or a modified GLB intervention, which included the provision of LGx information and advice (GLB+LGx). There were no costs associated with enrollment in either of the interventions for the participants. Computer-generated randomization [16] was conducted by the first author using randomly permuted blocks and group allocation was concealed for participants during a two-week run-in period. Comprehensive study methods have been previously published elsewhere [17] and are more briefly detailed below. 

### 2.1. Participants and Setting 

This study took place at the East Elgin Family Health Team, a primary care clinic in Aylmer, Ontario, Canada. Participants enrolled in the GLB program were invited to participate in the study if they met the following inclusion criteria: BMI ≥25 kg/m^2^, ≥18 years of age, English-speaking, willing to undergo genetic testing, having access to a computer with internet at least one day per week and not seeing another healthcare provider for weight loss advice outside of the study. Pregnancy and lactation were considered exclusion criteria. The first author was responsible for enrolling participants. A sample size was calculated based on the primary outcome indicating that a total of 74 participants were needed (*n* = 37 per group) for this trial.

### 2.2. Interventions 

The description of interventions was guided by the Template for Intervention Description and Replication Checklist. The first author (JRH), a registered dietitian specializing in nutrigenomics and a trained lifestyle coach for the GLB program, was responsible for running all interventions. Participants selected a GLB group at the East Elgin Family Health Team in Aylmer, Ontario, Canada that best suited their schedule and were blinded to the group assignments at this time (during the run-in). The average group size was 14 participants and the complete curriculums and intervention procedures have been previously detailed in-depth elsewhere [17]. Both group interventions were 12 months in duration, consisting of 23 group-based sessions and 3 one-on-one sessions. Interventions aimed to assist participants with weight management and healthy lifestyle change, with particular focuses on nutrition and PA. The GLB intervention was based on population lifestyle guidelines and the GLB+LGx intervention was based on genetically tailored lifestyle advice; this was the only difference between the two group interventions. To improve intervention adherence, participants were given reminder calls for their one-on-one appointments and for the start of their GLB group program. 

The five randomized standard GLB groups (*n* = 70) followed the established GLB program curriculum [18] in which participants were given population-based information and advice, while focusing on following a calorie-controlled, moderate-fat (25% of calories) nutrition plan with at least 150 min of weekly moderate-intensity PA. Participants were also provided with a 1-page summary report of their nutrition and PA guidelines at the first group meeting, which outlined population-based targets including acceptable macronutrient distribution ranges [19] for: protein, total fat, saturated fat, monounsaturated fat, polyunsaturated fat, sodium, calories, snacking and physical activity. The five randomized modified GLB+LGx groups (*n* = 70) followed a modified GLB program curriculum in which participants were given genetic-based information and advice, which differed from the advice given to the standard GLB group, while focusing on following a calorie-controlled nutrition plan. The nutrition and PA targets were personalized based on their individual genetic variation. For example, participants with the AA variant of *FTO* (rs9939609) were advised to engage in at least 30–60 minutes daily of physical activity six days per week with muscle-strengthening activities at least two days per week, rather than receiving the standard population-based advice to aim for 150 minutes weekly of physical activity with at least two days per week of muscle-strengthening activity. Participants were also provided with a 1-page summary report of their nutrition and PA guidelines at the first group meeting, which outlined genetic-based information and advice related to: protein, total fat, saturated fat, monounsaturated fat, polyunsaturated fat, sodium, calories, snacking and physical activity. Sample reports for both groups have been previously published [17]. As a point of reference (i.e., to compare their current intake to their recommended intake), participants were given the results of their baseline self-administered online food frequency questionnaire (The Past-Month Canadian Diet History Questionnaire II), which participants completed during the run-in period. There were three follow-up one-on-one appointments with a registered dietitian, which occurred after 3, 6 and 12 months. At these appointments, participants completed a self-administered TPB questionnaire and lifestyle recommendations were reviewed with a registered dietitian. All group-based GLB sessions and one-on-one appointments were conducted face-to-face.

### 2.3. Outcomes 

**Figure 2:**
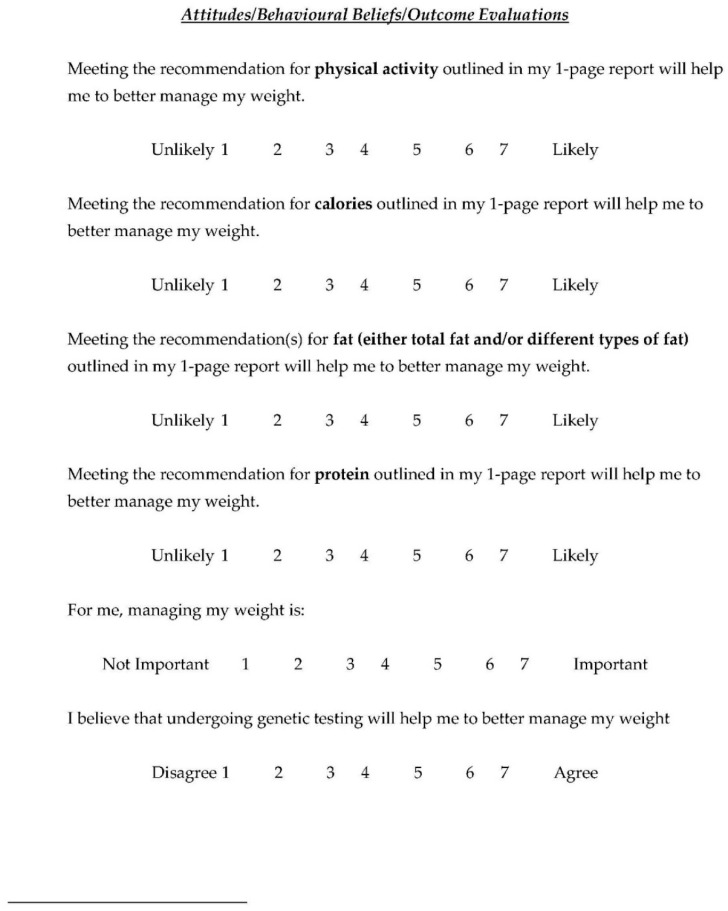

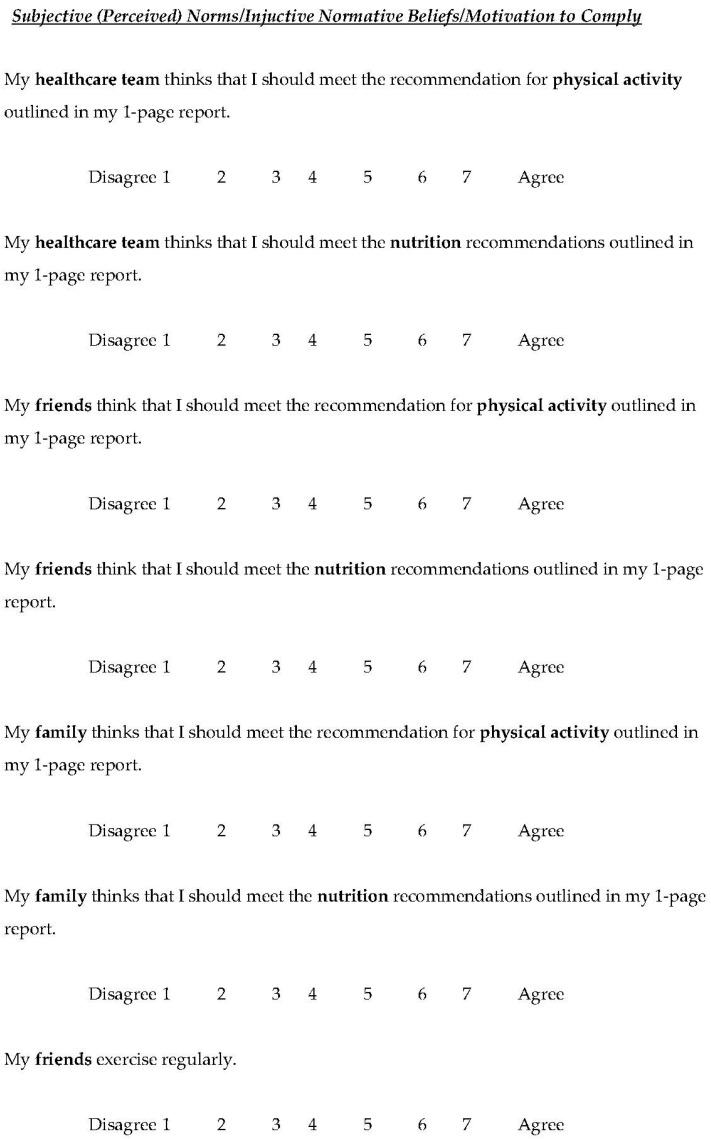

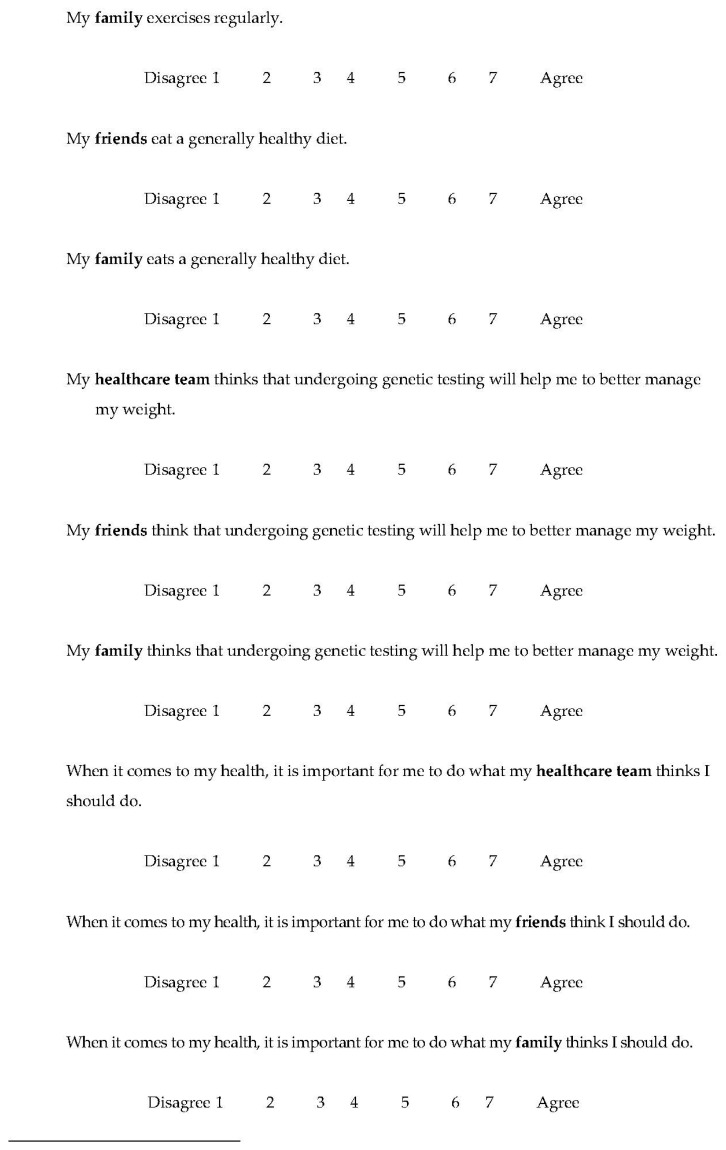

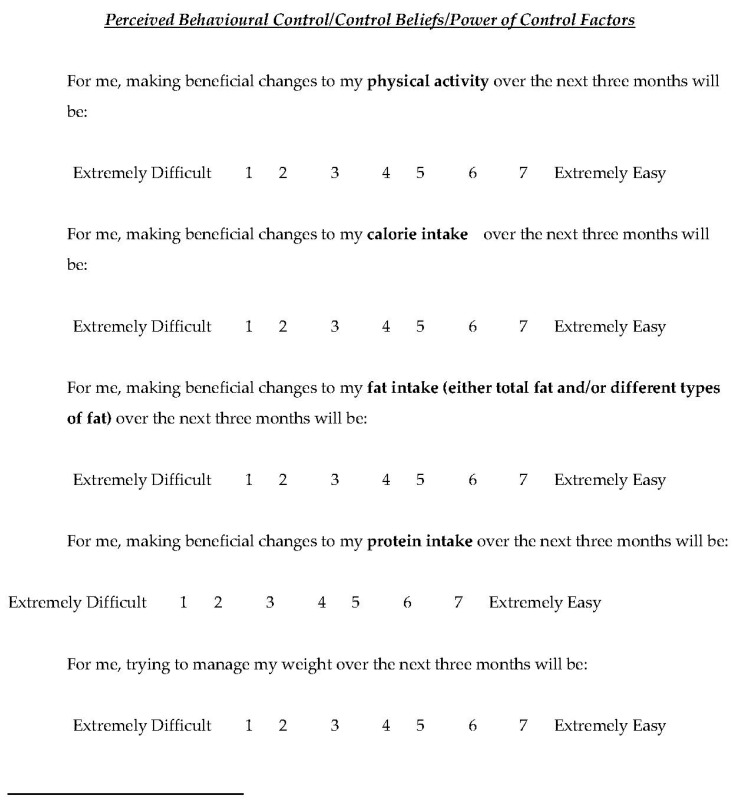

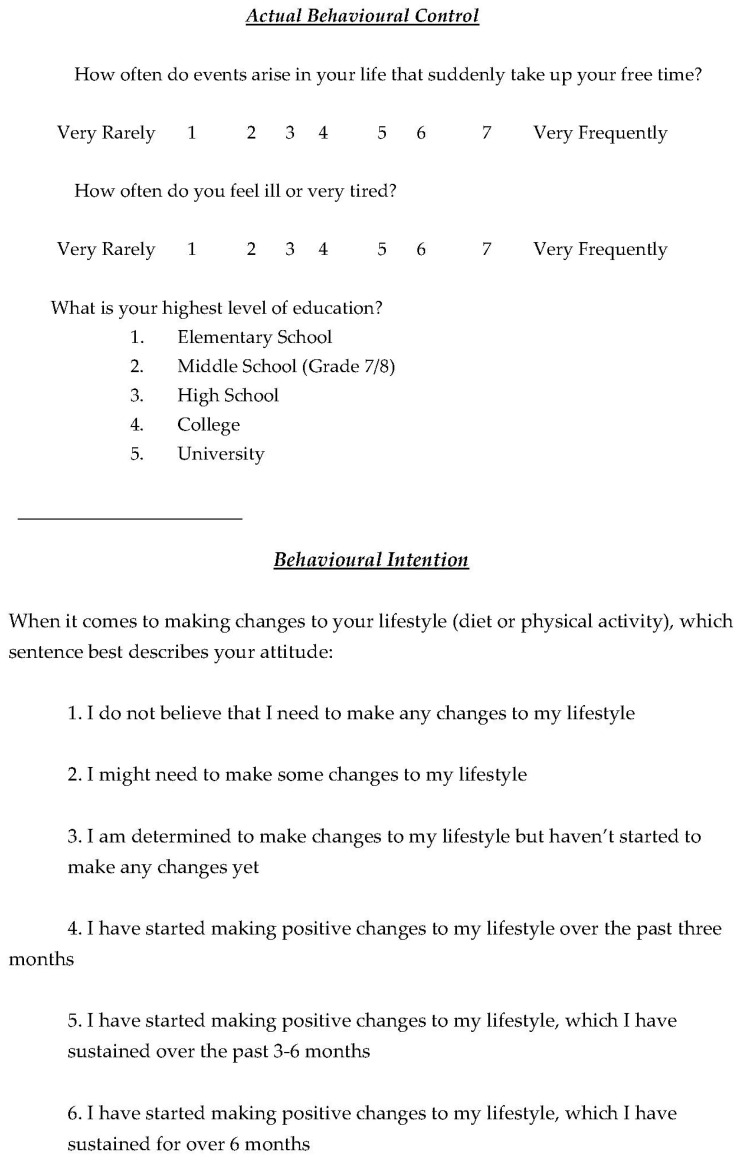
Select TPB Questions.

Change in the TPB components (attitudes, subjective norms and perceived behavioural control) was a pre-specified secondary outcome of the NOW trial. A TPB questionnaire was developed with guidance from Ajzen’s Guide to Constructing a TPB Questionnaire [3] and select TPB questions included in the present study are outlined in Figure 2. Attitudes, subjective norms and perceived behavioural control were measured on a Likert scale from 1 through 7. Stage of change was also measured, based on the Transtheoretical Model, using a Likert scale of 1 through 6. Actual behavioural control was measured through questions assessed on a Likert scale of 1 through 7, education was assessed categorically and income was assessed continuously. TPB questionnaires were administered at five time points: during a two-week run-in period, immediately after the first group session, where participants were given either a summary report of population-based guidelines or genetic-based guidelines, and at 3-, 6- and 12-month follow-ups. 

### 2.4. Statistical Analyses

Linear mixed models were used to conduct within- and between-group analyses using SPSS version 26.0, while controlling for measures of actual behavioural control. Self-reported measures of actual behavioural control, collected from participant surveys, included annual household income (CDN $), perceptions about events arising in one’s day-to-day life that suddenly take up one’s free time, perceptions about the frequency of feeling ill or tired and highest achieved level of education. All analyses were intention-to-treat by originally assigned groups with mean value imputation conducted for missing data. A Bonferroni correction for multiple testing was used.

## 3. Results

**Figure 3:**
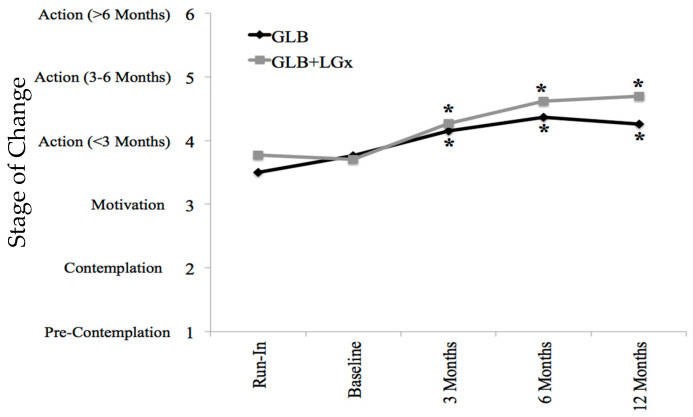
Stages of change by time point and group. Stage of change was measured on a Likert scale of 1 through 6 based on the transtheoretical model (stages of change); 1 represented pre-contemplation, 2 represented contemplation, 3 represented motivation, 4 represented action (<3 months), 5 represented action (for 3–6 months) and 6 represented maintenance (>6 months). Asterisks indicate significant differences from run-in (pre-intervention), within groups: *p* ≤ 0.001 at all time points in both groups. GLB (*n* = 70 with mean value imputation): Run-In 3.50 ± 0.18 (mean ± SE), 95% CI: 3.10–3.82; Baseline 3.76 ± 0.18, 95% CI: 3.41–4.12; 3 Months* 4.15 ± 0.15, 95% CI: 3.85–4.44; 6 Months* 4.37 ± 0.17, 95% CI: 4.03–4.70; 12 Months* 4.26 ± 0.19, 95% CI: 3.89–4.63. GLB+LGx (*n* = 70 with mean value imputation): Run-In 3.77 ± 0.16 (mean ± SE), 95% CI: 3.46–4.09; Baseline 3.70 ± 0.15, 95% CI: 3.40–4.00; 3 Months* 4.27 ± 0.12, 95% CI: 4.02–4.51; 6 Months* 4.62 ± 0.14, 95% CI: 4.33–4.90; 12 Months* 4.70 ± 0.16, 95% CI: 4.39–5.02. GLB: Group Lifestyle Balance™; LGx: lifestyle genomics.

Table 1, Table 2 and Table 3 outline the specific changes in the TPB components across groups at each of the five time points. Participants were highly motivated at baseline and there were significant increases in stages of change from the run-in to 3-, 6- and 12-month follow-up in both the GLB and GLB+LGx groups. This is further depicted in Figure 3.

### 3.1. Summary of Sample and Baseline Data 

As previously published [15], the sample consisted of primarily middle-aged, middle income, Caucasian females and there were no unintended harms reported in this RCT. Recruitment ended after enough participants had been recruited for ten GLB/GLB+LGx groups. Baseline descriptive data (Table 1, Table 2 and Table 3) indicate that participants had positive attitudes towards the effectiveness of nutrition and PA for weight management, and reported that weight management was highly important to them. They also perceived that undergoing genetic testing would assist with weight management. Participants had overall neutral subjective norms related to friends and family consuming a healthy diet and engaging in PA, but perceived that their friends, family and healthcare team (HCT) believed that it was important for them to achieve their nutrition and PA recommendations. With respect to perceptions about friends’, families’ and their HCTs’ beliefs about the effectiveness of genetic testing for weight management, participants overall perceived that their HCT believed that genetic testing could assist with weight management, while perceptions on this matter from friends and family were lesser, but still trended towards positive beliefs. The reported importance of following lifestyle advice from friends and family was negative to neutral, while the importance of following such advice from participants’ HCT was positive. Baseline measures of perceived behavioural control were overall neutral, with baseline stage of change between “motivation” and “action” (short-term; <3 months).

### 3.2. Differences between GLB and GLB+LGx Groups

In all cases of between-group differences (for both significant (*p* < 0.05) and approaching significant (*p* = 0.05–0.06) values) for changes in the TPB components, the standard GLB group exhibited reductions, whereas the GLB+LGx group exhibited increases (improvements). Significant differences were observed for changes between groups at 3 months for subjective norms related to the perception that friends believed that LGx would help with weight management. Moreover, there were significant differences between groups at 12 months for subjective norms related to the perception that family believed that it was important for the participant to achieve the PA recommendations. Several between-group differences trended towards significance with *p* = 0.05–0.06. After 3 months, changes in attitudes towards the effectiveness of LGx for weight management differed between groups, favouring the GLB+LGx group. Differences were also observed between groups at 12 months for changes in subjective norms; participants perceived that family members believed that it was important for the participant to achieve their physical activity (*p* < 0.05) and nutrition recommendations (*p* = 0.05–0.06), with greater improvements in these subjective norm scores in the GLB+LGx group compared to the standard GLB group. Furthermore, changes in perceptions about family members believing that genetic testing would help with weight management differed between the GLB and GLB+LGx group at 12 months (*p* = 0.05–0.06). 

### 3.3. Change in Attitudes Within Groups

Overall, significant changes (improvements) in attitudes towards the effectiveness of the nutrition and PA recommendations for weight management were similar in the GLB and GLB+LGx groups, though changes tended to be more long-term in the GLB+LGx group. 

### 3.4. Change in Subjective Norms Within Groups

Changes in subjective norms also tended to be more long-term in the GLB+LGx group, with significant changes in five subjective norm measures observed in this group, occurring after the 6- and 12-month follow-ups. Notably, there were significant long-term changes in subjective norms related to both friends and family consuming a healthy diet. By comparison, the standard GLB group exhibited three significant subjective norm changes, occurring shorter term after the 3- and 6-month follow-ups.

### 3.5. Change in Perceived Behavioural Control 

Both interventions appear to have affected measures of perceived behavioural control over both the short term and the long term. There were increases in the perceived difficulty of changing protein intake in the standard GLB group, but not the GLB+LGx group.

## 4. Discussion

Overall, our results indicate that the provision of genetically tailored lifestyle information and advice tended to impact antecedents of behaviour change, more so over the long term through within-group analyses while population-based advice tended to impact antecedents of behaviour change more over the short term through within-group analyses (e.g., attitudes towards dietary fat intake, perceptions that friends and family consume a healthy diet and perceptions about the impact of genetic-based advice for weight management). These findings directly relate to previously published NOW trial results. First, we found that the addition of LGx information and advice motivated participants to improve their diet to a significantly greater extent than population-based advice alone after 12-month follow-up [20]. In particular, the GLB+LGx group significantly reduced their dietary fat intake and better adhered to dietary fat guidelines compared to the standard GLB group [20]. The results of the TPB analysis indicate that these dietary fat changes may have extended to friends and family (social norms) as evidenced by the improvement in scores related to friends and family eating a healthy diet among the GLB+LGx group after 6 and 12 months. Future research should explore this phenomenon further. In addition, our previous finding that the GLB+LGx group exhibited significantly greater improvements in body composition after 3- and 6-month follow-ups [15] relates to the current report’s finding that attitudes towards the effectiveness of genetic-based advice for weight management were higher in the GLB+LGx group after 3 months (approaching significance). This also relates to the finding that friends and family had enhanced perceptions compared to baseline about the efficacy of genetic testing to assist with weight management after 3 and 6 months in the GLB+LGx group. The increase in perceived behavioural control for perceived difficulty changing protein intake in the GLB group, but not the GLB+LGx group is interesting given that there was a higher and more difficult to achieve protein targets (25–35% of calories) given to participants with the AA variant of the *FTO* genotype (rs9939609) [17]. In the standard GLB group, all participants were advised to consume 10–35% of calories from protein. There were also a number of changes in TPB determinants that were similar in the GLB and GLB+LGx groups, such as increases in the perceived difficulties about changing physical activity, calories and managing weight overall. These findings demonstrate that these may be more challenging behaviours for participants to engage in and outcomes to achieve, regardless of the type of information/advice provided. Future research should continue to explore how to help patients enrolled in weight management programs overcome these challenges.

The results of this study can also help to explain findings in the body of research exploring nutrition and PA behaviour change resulting from genetic testing, as recently detailed in a systematic review of LGx and lifestyle behaviour change [11]. Some studies have demonstrated that genetic testing helps motivate lifestyle behaviour change [8,9,10], whereas, in contrast, others have not [21,22,23], although one of these studies still reported positive changes in attitudes and perceptions towards omega-3 in the genetic group [22]. Interestingly, there have been only four long-term (12-month) RCTs assessing changes in nutrition in genetic interventions compared to population-based interventions [8,9,11,20,24]. All of these RCTs have demonstrated that long-term nutrition-related behaviour change was greater with the addition of genetic-based information/advice [8,9,20,24]. Thus, we report a potential explanation of these long-term findings, guided by the TPB. It appears that antecedents of behaviour change tend to be positively impacted more long term in the GLB+LGx intervention group, while changes in the standard GLB group tend to be more short term, based on the within-group analyses. Moreover, between-group differences in subjective norms observed at 12-month follow-up suggest that social pressures and norms may be influencing long-term changes in lifestyle habits. Therefore, the findings from the present study provide a potential theoretical explanation to help us to understand results from the current body of genetic testing behaviour change research. Indeed, population-based research consistently indicates that nutrition interventions impact short-term dietary changes, but long-term dietary changes remain challenging [25,26]. This has been referred to as “the adherence problem” [25]. Nutrigenetic interventions could help to mitigate this problem.

Furthermore, it was interesting to find that there were long-term improvements in perceptions that friends and family eat a healthy diet, but only in the GLB+LGx group. Thus, we speculate that it is possible that long-term behaviour change motivated by genetic testing could affect not only the individual undergoing genetic testing, but could expand beyond the individual into their social groups. This speculation is supported in the literature, with numerous studies indicating the significant impact of social modeling and social norms on food intake [27,28,29,30]. Future LGx interventional research should seek to explore this phenomenon further.

There are some limitations that should be noted. Intention-to-treat analyses (while still considered the gold-standard analyses for RCTs) tend to be more conservative than other methods of analysis [31,32], therefore actual changes over time may have been underestimated in the present study. In addition, generalizability is limited to middle-aged females enrolled in a lifestyle change weight management program with an average household income of approximately $73,000 CDN. Future research should seek to replicate the current study in more diverse study populations, with health-related outcomes of interest beyond weight management. Moreover, research suggests that eating healthfully and being physically active are less common for people of lower socioeconomic status compared to those of higher socioeconomic status [33,34]. It is thus possible that populations with higher income may be able to make more lifestyle changes following the provision of LGx testing given their higher food budget (and vice versa for lower income), and thus the TPB constructs may be affected differently depending on income. Future research should also explore this. It should be further noted that the income of genetic testing consumers in a pragmatic setting may be higher than those enrolled in funded research studies (who are typically not responsible for covering the cost of tests) given that they must purchase a test, often costing hundreds of dollars [35]. Therefore, income disparities likely exist for those who can (and cannot) afford to undergo LGx testing. This is a topic warranting further discussion.

Given that this is the first study exploring the potential theoretical mechanism for behaviour change resulting from personalized genetic interventions, research should continue to explore this topic further. TPB or other theory-based questionnaires can be added to genetic-based interventional studies assessing lifestyle behaviour change. These studies should include long-term follow-up of at least one year, given that it appears that changes in determinants of behaviour change and actual behaviour change resulting from genetic testing interventions tend to be more long-term [8,9,20,24], and that strategies are needed to solve the “adherence problem” [25]. This research should also explore which particular aspects of genetic interventions are the most motivational, and how subjective norms impact behaviour change (or lack thereof).

Overall, the present work demonstrates that the TPB can help to explain the results from studies exploring the impact of genetic testing on lifestyle behaviour change. These results provide a potential theoretical explanation, demonstrating that LGx interventions tend to impact antecedents of behaviour change more long-term, with changes in certain subjective norms being significantly greater with a LGx-guided intervention compared to a population-based intervention. In conclusion, this study supports the hypothesis that the TPB can help to explain why genetically tailored lifestyle information and advice can lead to improvements in lifestyle behaviour change.

## Figures and Tables

**Table 1:** Change in attitudes by intervention group over 12-month follow-up.

Group	Lifestyle Factor and TPB Component	Run-In (Mean ± SE, 95% CI)	Baseline (Mean ± SE, 95% CI)	3 Months (Mean ± SE, 95% CI)	6 Months (Mean ± SE, 95% CI)	12 Months (Mean ± SE, 95% CI)	Between-Group Analyses
**GLB**	Attitudes towards PA for weight management	5.79 ± 0.22, 5.82–6.52	6.20 ± 0.21,	**6.21 ± 0.18,**	**6.33 ± 0.17,**	**6.17 ± 0.18,**	NS
			5.80–6.61	**5.85–6.57**	**5.98–6.67**	**5.82–6.65**	
			(*p* = 0.06)	(***p*** **= 0.04)**	(***p*** **< 0.01)**	(***p*** **= 0.03)**	
**GLB+LGx**		5.89 ± 0.20, 5.50–6.28	**6.51 ± 0.14,**	**6.50 ± 0.15,**	**6.63 ± 0.11,**	**6.55 ± 0.11,**	
			**6.23–6.80**	**6.20–6.81**	**6.42–6.85**	**6.34–6.76**	
			**(** ***p*** **< 0.01)**	(***p*** **< 0.01)**	(***p*** **< 0.01)**	(***p*** **< 0.01)**	
**GLB**	Attitudes towards kcal for weight management	6.34 ± 0.15, 6.05–6.64	6.44 ± 0.15,	6.27 ± 0.16,	6.41 ± 0.14,	6.36 ± 0.14,	NS
			6.15–6.74	5.95–6.59	6.13–6.70	6.09–6.64	
			(*p* = 0.475)	(*p* = 0.627)	(*p* = 0.583)	(*p* = 0.860)	
**GLB+LGx**		6.28 ± 0.14, 6.00–6.56	6.44 ± 0.12,	6.55 ± 0.11,	6.53 ± 0.10,	6.49 ± 0.09,	
			6.21–6.68	6.32–6.77	6.34–6.73	6.31–6.67	
			(*p* = 0.27)	(*p* = 0.07)	(*p* = 0.05)	(*p* = 0.07)	
**GLB**	Attitudes towards dietary fat for weight management	6.11 ± 0.18, 5.76–6.45	**6.48 ± 0.13,**	6.24 ± 0.15,	6.36 ± 0.12,	6.37 ± 0.12,	NS
			**6.23–6.74**	5.96–6.54	6.13–6.61	6.13–6.61	
			**(** ***p*** **= 0.03)**	(*p* = 0.45)	(*p* = 0.13)	(*p* = 0.10)	
**GLB+LGx**		6.19 ± 0.15, 5.90–6.49	6.42 ± 0.14,	**6.50 ± 0.12,**	**6.48 ± 0.12**	6.39 ± 0.12,	
			6.14–6.69	**6.25–6.74**	**6.23–6.72**	6.13–6.62	
			(*p* = 0.15)	**(** ***p*** **= 0.03)**	(***p*** **= 0.04)**	(*p* = 0.14)	
**GLB**	Attitudes towards protein for weight management	5.97 ± 0.16, 5.65–6.29	**6.44 ± 0.14,**	6.13 ± 0.15,	**6.30 ± 0.12,**	6.23 ± 0.13,	NS
			**6.16–6.71**	5.84–6.42	**6.06–6.54**	5.98–6.49	
			**(** ***p*** **< 0.01)**	(*p* = 0.34)	**(** ***p*** **= 0.02)**	(*p* = 0.05)	
**GLB+LGx**		6.25 ± 0.15, 5.96–6.54	6.50 ± 0.13,	**6.54 ± 0.11,**	**6.58 ± 0.09,**	6.48 ± 0.10,	
			6.25–6.76	**6.32–6.75**	**6.40–6.77**	6.28–6.67	
			(*p* = 0.12)	**(** ***p*** **< 0.05)**	(***p*** **= 0.01)**	(*p* = 0.07)	
**GLB**	Attitudes towards the importance of weight management	6.67 ± 0.12, 6.43–6.92	6.63 ± 0.12,	6.67 ± 0.10,	6.61 ± 0.11,	6.60 ± 0.11,	NS
			6.40–6.89	6.46–6.87	6.39–6.83	6.39–6.82	
			(*p* = 0.73)	(*p* = 0.98)	(*p* = 0.57)	(*p* = 0.43)	
**GLB+LGx**		6.78 ± 0.11, 6.57–6.96	6.90 ± 0.08,	6.72 ± 0.10,	6.76 ± 0.08,	6.68 ± 0.09,	
			6.75–7.05	6.52–6.93	6.60–6.92	6.50–6.89	
			(*p* = 0.22)	(*p* = 0.59)	(*p* = 0.82)	(*p* = 0.22)	
**GLB**	Attitudes towards the impact of undergoing genetic testing to enhance weight management	6.31 ± 0.22, 5.88–6.74	6.16 ± 0.22,	6.04 ± 0.21,	6.16 ± 0.18,	6.09 ± 0.18,	* *p* = 0.06 at 3 months (AS)
			5.73–6.58	5.63–6.44	5.81–6.52	5.73–6.45	
			(*p* = 0.48)	(*p* = 0.19) *	(*p* = 0.42)	(*p* = 0.18)	
**GLB+LGx**		6.35 ± 0.16, 6.04–6.67	6.65 ± 0.14,	6.58 ± 0.13,	6.58 ± 0.12,	6.46 ± 0.13,	
			6.39–6.92	6.33–6.83	6.35–6.82	6.21–6.71	
			(*p* = 0.07)	(*p* = 0.14) *	(*p* = 0.12)	(*p* = 0.44)	

Attitudes were measured on a Likert scale of 1 (unlikely) through 7 (likely); 4 was considered neutral. Bolded values are significantly different from run-in (pre-intervention), within groups. GLB (*n* = 70 with mean value imputation); GLB+LGx (*n* = 70 with mean value imputation). * Significant or approaching significant differences for changes between groups. TPB: Theory of Planned Behaviour; NS: non-significant; AS: approaching significance; PA: physical activity; kcal: calories; GLB: Group Lifestyle Balance™; LGx: lifestyle genomics.

**Table 2:** Change in subjective norms by intervention group over 12-month follow-up.

Group	Lifestyle Factor and TPB Component	Run-In (Mean ± SE, 95% CI)	Baseline (Mean ± SE, 95% CI)	3 Months (Mean ± SE, 95% CI)	6 Months (Mean ± SE, 95% CI)	12 Months (Mean ± SE, 95% CI)	Between-Group Analyses
**GLB**	Subjective norms: HCT and PA	5.90 ± 0.25, 5.40–6.40	6.11 ± 0.21,	6.15 ± 0.21,	6.23 ± 0.20,	6.08 ± 0.21,	NS
			5.70–6.52	5.74–6.56	5.84–6.61	5.66–6.50	
			(*p* = 0.36)	(*p* = 0.27)	(*p* = 0.12)	(*p* = 0.36)	
**GLB+LGx**		6.25 ± 0.22, 5.82–6.68	6.08 ± 0.24,	6.41 ± 0.20,	6.45 ± 0.18,	6.30 ± 0.20,	
			5.61–6.55	6.02–6.81	6.09–6.81	5.91–6.69	
			(*p* = 0.45)	(*p* = 0.35)	(*p* = 0.16)	(*p* = 0.69)	
**GLB**	Subjective norms: HCT and nutrition	6.71 ± 0.17, 5.84–6.50	6.25 ± 0.15,	6.29 ± 0.15,	6.42 ± 0.13,	6.28 ± 0.14,	NS
			5.95–6.56	6.00–6.59	6.16–6.68	6.00–6.56	
			(*p* = 0.619)	(*p* = 0.419)	(*p* = 0.061)	(*p* = 0.372)	
**GLB+LGx**		6.39 ± 0.16, 6.06–6.71	6.18 ± 0.19,	6.55 ± 0.14,	6.59 ± 0.13,	6.43 ± 0.16,	
			5.80–6.57	6.27–6.83	6.32–6.85	6.12–6.75	
			(*p* = 0.29)	(*p* = 0.24)	(*p* = 0.09)	(*p* = 0.68)	
**GLB**	Subjective norms: Friends and PA	5.36 ± 0.26, 4.85–5.87	5.15 ± 0.25,	5.06 ± 0.24,	5.35 ± 0.22,	5.02 ± 0.22,	NS
			4.65–5.65	4.60–5.53	4.83–5.68	4.58–5.87	
			(*p* = 0.44)	(*p* = 0.25)	(*p* = 0.66)	(*p* = 0.16)	
**GLB+LGx**		5.28 ± 0.25, 4.80–5.77	5.15 ± 0.27,	5.58 ± 0.22,	5.53 ± 0.21,	5.37 ± 0.22,	
			4.61–5.70	5.15–6.00	5.11–5.95	4.94–5.80	
			(*p* = 0.64)	(*p* = 0.19)	(*p* = 0.24)	(*p* = 0.65)	
**GLB**	Subjective norms: Friends and nutrition	5.43 ± 0.23, 4.96–5.89	5.33 ± 0.22,	5.28 ± 0.21,	5.38 ± 0.19,	5.09 ± 0.21,	NS
			4.90–5.76	4.87–5.70	5.00–5.78	4.68–5.49	
			(*p* = 0.71)	(*p* = 0.55)	(*p* = 0.83)	(*p* = 0.13)	
**GLB+LGx**		5.42 ± 0.23, 4.96–5.88	5.07 ± 0.27,	5.57 ± 0.21,	5.54 ± 0.20,	5.48 ± 0.21,	
			4.54–5.60	5.15–5.99	5.14–5.94	5.06–5.89	
			(*p* = 0.19)	(*p* = 0.47)	(*p* = 0.52)	(*p* = 0.74)	
**GLB**	Subjective norms: Family and PA	5.86 ± 0.23, 5.40–6.31	5.85 ± 0.24,	5.69 ± 0.22,	5.85 ± 0.20,	5.57 ± 0.22,	* ***p*** **= 0.049 at 12 months**
			5.39–6.32	5.25–6.12	5.45–6.25	5.13–6.31	
			(*p* = 0.99)	(*p* = 0.41)	(*p* = 0.98)	(*p* = 0.11) *	
**GLB+LGx**		5.81 ± 0.22, 5.37–6.24	5.57 ± 0.23,	5.89 ± 0.19,	6.08 ± 0.16,	6.01 ± 0.16,	
			5.12–6.03	5.51–6.27	5.76–6.40	5.70–6.33	
			(*p* = 0.37)	(*p* = 0.71)	(*p* = 0.17)	(*p* = 0.24) *	
**GLB**	Subjective norms: Family and nutrition	5.79 ± 0.20, 5.39–6.18	5.81 ± 0.21,	5.65 ± 0.20,	5.80 ± 0.18,	5.47 ± 0.20,	* *p* = 0.050 at 12 months(AS)
			5.40–6.21	5.25–6.05	5.45–6.16	5.07–5.86	
			(*p* = 0.916)	(*p* = 0.473)	(*p* = 0.907)	(*p* = 0.058) *	
**GLB+LGx**		5.93 ± 0.22, 5.50–6.36	5.70 ± 0.22,	6.03 ± 0.18,	6.05 ± 0.17,	6.07 ± 0.16,	
			5.25–6.14	5.67–6.39	5.72–6.39	5.75–6.39	
			(*p* = 0.344)	(*p* = 0.631)	(*p* = 0.523)	(*p* = 0.387) *	
**GLB**	Perception that friends exercise regularly	3.92 ± 0.32, 3.29–4.56	4.33 ± 0.31,	4.04 ± 0.28,	4.29 ± 0.27,	4.15 ± 0.27,	NS
			3.73–4.94	3.48–4.56	3.76–482	3.61–4.69	
			(*p* = 0.16)	(*p* = 0.66)	(*p* = 0.12)	(*p* = 0.27)	
**GLB+LGx**		3.87 ± 0.28, 3.23–4.42	3.99 ± 0.28,	3.89 ± 0.26,	4.14 ± 0.24,	4.10 ± 0.25,	
			3.44–4.54	3.38–4.40	3.66–4.61	3.61–4.59	
			(*p* = 0.68)	(*p* = 0.95)	(*p* = 0.27)	(*p* = 0.30)	
**GLB**	Perception that family exercises regularly	3.71 ± 0.35, 3.02–4.41	4.02 ± 0.33,	3.91 ± 0.32,	**4.26 ± 0.31,**	4.07 ± 0.32,	NS
			3.36–4.68	3.27–4.55	**3.65–4.87**	3.43–4.71	
			(*p* = 0.28)	(*p* = 0.45)	**(** ***p*** **= 0.02)**	(*p* = 0.08)	
**GLB+LGx**		4.21 ± 0.30, 3.61–4.81	4.55 ± 0.30,	4.60 ± 0.26,	4.55 ± 0.27,	4.26 ± 0.26,	
			3.97–5.14	4.07–5.12	4.01–5.09	3.73–4.78	
			(*p* = 0.23)	(*p* = 0.12)	(*p* = 0.15)	(*p* = 0.81)	
**GLB**	Perception that friends eat a healthy diet	3.94 ± 0.30, 3.35–4.52	4.26 ± 0.28,	4.21 ± 0.26,	4.34 ± 0.26,	4.28 ± 0.26,	NS
			3.72–4.81	3.70–4.73	3.83–4.86	3.77–4.80	
			(*p* = 0.20)	(*p* = 0.23)	(*p* = 0.06)	(*p* = 0.05)	
**GLB+LGx**		4.43 ± 0.20, 4.03–4.83	4.43 ± 0.21,	4.73 ± 0.20,	**4.83 ± 0.17,**	**4.84 ± 0.18,**	
			4.02–4.85	4.34–5.12	**4.51–5.16**	**4.49–5.19**	
			(*p* = 0.99)	(*p* = 0.17)	**(** ***p*** **= 0.03)**	(***p*** **= 0.03)**	
**GLB**	Perception that family eats a healthy diet	4.64 ± 0.30, 4.05–5.23	4.66 ± 0.30,	**5.06 ± 0.26,**	5.02 ± 0.26,	4.84 ± 0.27,	NS
			4.07–5.25	**4.54–5.59**	4.51–5.54	4.31–5.37	
			(*p* = 0.93)	**(** ***p*** **< 0.05)**	(*p* = 0.05)	(*p* = 0.22)	
**GLB+LGx**		4.95 ± 0.21, 4.53–5.38	5.02 ± 0.22,	5.23 ± 0.21,	**5.42 ± 0.19,**	**5.32 ± 0.19,**	
			4.58–5.45	4.81–5.64	**5.04–5.80**	**4.94–5.70**	
			(*p* = 0.76)	(*p* = 0.17)	**(** ***p*** **< 0.01)**	(***p*** **= 0.01)**	
**GLB**	Perception that HCT believes that genetic testing will help with weight management	5.72 ± 0.23, 5.27–6.17	5.63 ± 0.23,	5.59 ± 0.21,	5.72 ± 0.19,	5.67 ± 0.20,	NS
			5.18–6.09	5.19–6.00	5.34–6.09	5.27–6.07	
			(*p* = 0.72)	(*p* = 0.55)	(*p* = 0.98)	(*p* = 0.81)	
**GLB+LGx**		6.11 ± 0.20, 5.72–6.50	5.91 ± 0.22,	6.36 ± 0.16,	6.32 ± 0.15,	6.06 ± 0.20,	
			5.48–6.34	6.04–6.67	6.02–6.63	5.67–6.45	
			(*p* = 0.37)	(*p* = 0.15)	(*p* = 0.17)	(*p* = 0.77)	
**GLB**	Perception that friends believe that genetic testing will help with weight management	4.93 ± 0.29, 4.35–5.51	4.66 ± 0.29,	**4.38 ± 0.27,**	4.80 ± 2.54,	4.53 ± 0.26,	* ***p*** **= 0.024 at 3 months**
			4.10–5.23	**3.84–4.92**	4.30–5.31	4.01–5.05	
			(*p* = 0.34)	**(** ***p*** = 0.04) *	(*p* = 0.59)	(*p* = 0.06)	
**GLB+LGx**		4.92 ± 0.27, 4.39–5.46	4.71 ± 0.28,	5.18 ± 0.24,	5.24 ± 0.23,	4.99 ± 0.22,	
			4.15–5.23	4.71–5.66	4.79–5.68	4.55–5.43	
			(*p* = 0.46)	(*p* = 0.28) *	(*p* = 0.15)	(*p* = 0.71)	
**GLB**	Perception that family believes that genetic testing will help with weight management	5.16 ± 0.29, 4.59–5.72	4.92 ± 0.27,	4.86 ± 0.25,	5.13 ± 0.23,	4.81 ± 0.25,	* *p* = 0.05 at 12 months (AS)
			4.39–5.45	4.37–5.35	4.68–5.58	4.31–5.31	
			(*p* = 0.40)	(*p* = 0.26)	(*p* = 0.91)	(*p* = 0.14) *	
**GLB+LGx**		5.16 ± 0.25, 4.65–5.66	5.26 ± 0.25,	5.50 ± 0.22,	**5.67 ± 0.21,**	5.40 ± 0.19,	
			4.76–5.75	5.06–5.94	**5.27–6.08**	5.03–5.77	
			(*p* = 0.72)	(*p* = 0.16)	**(** ***p*** **= 0.02)**	(*p* = 0.19) *	
**GLB**	Importance of following HCT’s advice	6.31 ± 0.16, 5.99–6.62	6.08 ± 0.17,	6.32 ± 0.14,	6.41 ± 0.12,	6.38 ± 0.12,	NS
			5.74–6.41	6.05–6.59	6.18–6.65	6.15–6.61	
			(*p* = 0.20)	(*p* = 0.95)	(*p* = 0.41)	(*p* = 0.53)	
**GLB+LGx**		6.52 ± 0.14, 6.25–6.79	6.52 ± 0.13,	6.55 ± 0.13,	6.66 ± 0.10,	6.50 ± 0.14,	
			6.25–6.78	6.29–6.81	6.45–6.86	6.22–6.78	
			(*p* = 0.96)	(*p* = 0.86)	(*p* = 0.25)	(*p* = 0.87)	
**GLB**	Importance of following friends’ advice	3.19 ± 0.34, 2.51–3.86	3.50 ± 0.34,	3.43 ± 0.32,	3.41 ± 0.31,	3.40 ± 0.30,	NS
			2.83–4.17	2.80–4.05	2.80–4.00	2.79–4.00	
			(*p* = 0.31)	(*p* = 0.39)	(*p* = 0.38)	(*p* = 0.33)	
**GLB+LGx**		3.49 ± 0.32, 2.85–4.13	3.38 ± 0.33,	3.50 ± 0.30,	3.62 ± 0.29,	3.71 ± 0.28,	
			2.72–4.04	2.91–4.10	3.05–4.20	3.15–4.28	
			(*p* = 0.74)	(*p* = 0.97)	(*p* = 0.63)	(*p* = 0.35)	
**GLB**	Importance of following family’s advice	4.70 ± 0.37, 3.97–5.44	4.94 ± 0.37,	4.79 ± 0.37,	4.65 ± 0.35,	4.65 ± 0.36,	NS
			4.21–5.67	4.06–5.51	3.95–5.36	3.95–5.36	
			(*p* = 0.42)	(*p* = 0.76)	(*p* = 0.80)	(*p* = 0.78)	
**GLB+LGx**		4.58 ± 0.39, 3.80–5.36	4.53 ± 0.37,	4.67 ± 0.34,	4.66 ± 0.33,	4.76 ± 0.32,	
			3.79–5.27	4.00–5.35	4.01–5.31	4.13–5.40	
			(*p* = 0.89)	(*p* = 0.78)	(*p* = 0.80)	(*p* = 0.47)	

Subjective norms were measured on a Likert scale of 1 (disagree) through 7 (agree); 4 was considered neutral. Bolded values are significantly different from run-in (pre-intervention), within groups. GLB (*n* = 70 with mean value imputation); GLB+LGx (*n* = 70 with mean value imputation). * Significant or approaching significant differences for changes between groups. TPB: Theory of Planned Behaviour; NS: non-significant; AS: approaching significance; HCT: healthcare team; kcal: calories; GLB: Group Lifestyle Balance™; LGx: lifestyle genomics.

**Table 3:** Change in perceived behavioural control by intervention group over 12-month follow-up.

Group	Lifestyle Factor and TPB Component	Run-In (Mean ± SE, 95% CI)	Baseline (Mean ± SE, 95% CI)	3-Months (Mean ± SE, 95% CI)	6-Month (Mean ± SE, 95% CI)	12-Months (Mean ± SE, 95% CI)	Between-Group Analyses
**GLB**	Perceived difficulty changing PA	4.05 ± 0.25, 4.09–5.07	4.19 ± 0.26,	**4.79 ± 0.24,**	**4.63 ± 0.22,**	**4.58 ± 0.25,**	NS
			3.68–4.70	**4.32–5.26**	**4.19–5.07**	**4.09–5.07**	
			(*p* = 0.60)	**(** ***p*** **< 0.01)**	(***p*** **< 0.01)**	(***p*** **= 0.02)**	
**GLB+LGx**		4.43 ± 0.24, 3.96–4.89	4.78 ± 0.24,	**5.02 ± 0.22,**	**5.01 ± 0.21,**	**5.15 ± 0.20,**	
			4.30–5.36	**4.58–5.46**	**4.58–5.43**	**4.75–5.55**	
			(*p* = 0.15)	**(** ***p*** **= 0.01)**	(***p*** **< 0.01)**	(***p*** **< 0.01)**	
**GLB**	Perceived difficulty changing kcal intake	4.16 ± 0.233	4.32 ± 0.22,	**4.81 ± 0.20,**	**4.82 ± 0.20,**	**4.63 ± 0.21,**	NS
			3.89–4.76	**4.42–5.19**	**4.44–5.19**	**4.22–5.03**	
			(*p* = 0.49)	**(** ***p*** **< 0.01)**	(***p*** **< 0.01)**	(***p*** **= 0.02)**	
**GLB+LGx**		4.32 ± 0.27, 3.78–4.86	4.49 ± 0.26,	4.74 ± 0.24,	**4.73 ± 0.23**	**4.68 ± 0.22,**	
			3.96–5.01	4.6–5.22	**4.27–5.18**	**4.24–5.12**	
			(*p* = 0.53)	(*p* = 0.07)	(***p*** **< 0.05)**	(***p*** **= 0.03)**	
**GLB**	Perceived difficulty changing dietary fat intake	4.35 ± 0.25, 3.86–4.84	4.33 ± 0.23,	**4.90 ± 0.20,**	**4.89 ± 0.20,**	4.64 ± 0.22,	NS
			3.88–4.79	**4.49–5.30**	**4.49–5.29**	4.21–5.08	
			(*p* = 0.94)	**(** ***p*** **= 0.01)**	(***p*** **= 0.01)**	(*p* = 0.16)	
**GLB+LGx**		4.44 ± 0.27, 3.90–4.97	4.20 ± 0.26,	4.75 ± 0.23,	**4.86 ± 0.23,**	4.75 ± 0.23,	
			3.68–4.72	4.30–4.72	**4.41–5.32**	4.28–5.21	
			(*p* = 0.37)	(*p* = 0.16)	(***p*** **= 0.04)**	(*p* = 0.08)	
**GLB**	Perceived difficulty changing protein intake	4.60 ± 0.24, 4.13–5.07	4.78 ± 0.22,	**5.12 ± 0.20,**	**5.04 ± 0.20,**	4.96 ± 0.21,	NS
			4.34–5.21	**4.72–5.51**	**4.64–5.43**	4.55–5.37	
			(*p* = 0.45)	**(** ***p*** **= 0.02)**	(***p*** **= 0.04)**	(*p* = 0.07)	
**GLB+LGx**		4.94 ± 0.25, 4.44–5.43	4.86 ± 0.22,	5.14 ± 0.20,	5.23 ± 0.20,	5.05 ± 0.21,	
			4.42–5.31	7.75–5.53	4.84–5.62	4.64–5.47	
			(*p* = 0.77)	(*p* = 0.37)	(*p* = 0.16)	(*p* = 0.53)	
**GLB**	Perceived difficulty managing weight	3.75 ± 0.24, 3.29–4.22	4.04 ± 0,23,	**4.38 ± 0.23,**	**4.44 ± 0.22,**	**4.39 ± 0.22,**	NS
			3.59–4.49	**3.92–4.83**	**3.98–4.89**	**3.95–4.83**	
			(*p* = 0.157)	**(** ***p*** **< 0.01)**	(***p*** **< 0.01)**	(***p*** **< 0.01)**	
**GLB+LGx**		4.10 ± 0.26, 3.59–4.60	4.12 ± 0.24,	**4.71 ± 0.22,**	**4.53 ± 0.24,**	**4.65 ± 0.22,**	
			3.65–4.59	**4.27–5.14**	**4.06–5.00**	**4.22–5.08**	
			(*p* = 0.925)	**(** ***p*** **< 0.01)**	(***p*** **= 0.04)**	(***p*** **< 0.01)**	

Perceived behavioural control was measured on a Likert scale of 1 (extremely difficult) through 7 (extremely easy); 4 was considered neutral. Bolded values are significantly different from run-in (pre-intervention), within groups. GLB (*n* = 70 with mean value imputation); GLB+LGx (*n* = 70 with mean value imputation). TPB: Theory of Planned Behaviour; NS: non-significant; PA: physical activity; kcal: calories; GLB: Group Lifestyle Balance™; LGx: lifestyle genomics.
